# The Pta-AckA Pathway Regulates LrgAB-Mediated Pyruvate Uptake in *Streptococcus mutans*

**DOI:** 10.3390/microorganisms8060846

**Published:** 2020-06-04

**Authors:** Sang-Joon Ahn, Shailja Desai, Min Lin, Kelly C. Rice

**Affiliations:** 1 Department of Oral Biology, College of Dentistry, University of Florida, Gainesville, FL 32610, USA; sdesai@dental.ufl.edu; 2 Department of Chemistry, College of Arts and Sciences, University of Florida, Gainesville, FL 32610, USA; linmin1@ufl.edu; 3 Department of Microbiology and Cell Science, Institute of Food and Agricultural Sciences, University of Florida, Gainesville, FL 32610, USA; kcrice@ad.ufl.edu

**Keywords:** *Streptococcus mutans*, LrgAB, pyruvate, acetyl-CoA, Pta, AckA

## Abstract

Pyruvate forms the central node of carbon metabolism and promotes growth as an alternative carbon source during starvation. We recently revealed that LrgAB functions as a stationary phase pyruvate uptake system in *Streptococcus mutans,* the primary causative agent of human dental caries, but its underlying regulatory mechanisms are still not clearly understood. This study was aimed at further characterizing the regulation of LrgAB from a metabolomic perspective. We utilized a series of GFP quantification, growth kinetics, and biochemical assays. We disclosed that LrgAB is critical for pyruvate uptake especially during growth under low-glucose stress. Inactivation of the Pta-Ack pathway, responsible for the conversion of acetyl-CoA to acetate, completely inhibits stationary phase *lrgAB* induction and pyruvate uptake, and renders cells insensitive to external pyruvate as a signal. Inactivation of Pfl, responsible for the conversion of pyruvate to acetyl-CoA under anaerobic conditions, also affected stationary phase pyruvate uptake. This study explores the metabolic components of pyruvate uptake regulation through LrgAB, and highlights its potential as a metabolic stimulator, contributing to the resuscitation and survival of *S. mutans* cells during nutritional stress.

## 1. Introduction

*Streptococcus mutans,* a primary contributor to human dental caries, is able to efficiently and rapidly adjust to carbohydrate limitation, which is essential for its pathogenic lifestyle, forming biofilms [1,2,3,4]. To cope with such an adverse environment, subpopulations of cells within the biofilm could enter low levels of metabolic phase [5,6,7,8] or undergo lysis or death [9,10] as strategies to survive and persist at a community level. In this context, interest in bacterial pyruvate flux and regulation is emerging, due to the following facts: pyruvate directs the key metabolic fluxes for growth and energy generation [11,12,13]; it is excreted as an overflow metabolite during growth and is reabsorbed during starvation [14,15,16,17]; it can promote growth of metabolically inactive stationary phase cells [18,19,20,21]; and it buffers external sources of oxidative stress, i.e., as an H_2_O_2_-scavenger [22,23,24,25]. These features of pyruvate have also been reported in *S. mutans* [26,27].

We recently revealed that the *S. mutans* LrgAB system—which is directly and positively regulated by the LytST two-component system (TCS) [28,29] and hypothesized to induce cell death and lysis with its partner operon CidAB—functions as a stationary phase pyruvate uptake system, with its activity modulated in response to glucose and oxygen levels [26]. More recently, we found that LrgAB pyruvate uptake activity is under the control of two global regulators, CcpA [30] and CodY [31], and is affected by the composition (i.e., acetate and potassium ion) of the growth medium [27]. This illustrates the metabolic complexity of LrgAB activity and pyruvate uptake regulation in *S. mutans.* However, from a metabolic standpoint, the regulation of LrgAB and pyruvate uptake is still not clearly understood.

In this study, we further characterize glucose dependence of the LrgAB system and disclose that pyruvate uptake via LrgAB is critical when *S. mutans* cells experience nutritionally low glucose stress. We also reveal that inactivation of the Pta-AckA pathway completely abolishes LrgAB activity, as well as the capability of the organism to take up environmental pyruvate. We further show that the Pfl pathway also contributes to *S. mutans* stationary phase pyruvate uptake. These findings suggest that LrgAB activity and pyruvate uptake are tightly controlled by pyruvate catabolic pathways and environmental metabolic conditions. Furthermore, given the capability of pyruvate to promote growth particularly under nutrient limitation, these data suggest that pyruvate may have the potential to limit the survival and persistence of the organism by interfering with its transport and metabolic fluxes.

## 2. Materials and Methods

### 2.1. Bacterial Strains and Growth Conditions

Strains and plasmids used in this study are listed in Table S1. *S. mutans* UA159 and its derivative strains were grown in brain heart infusion (BHI) medium (BD Difco™, Franklin, NJ, USA) as overnight static cultures at 37 °C in a 5% CO_2_ atmosphere. When necessary, antibiotics were added to cultures as follows: kanamycin (1 mg/mL) and spectinomycin (1 mg/mL). Overnight cultures were diluted 1:50 into fresh BHI broth, grown to an OD_600_≈0.4–0.5, and then used as seed cultures for growth kinetic and microplate reporter assays. The media used for the assays include BHI, chemically defined FMC [32,33] and TV (tryptone/vitamin) [34]. Except for BHI (already containing 11 mM glucose), FMC and TV media were supplemented by 11 mM glucose (named FMC11 and TV11, respectively) or different concentrations of glucose (named FMC2, FMC5, FMC7, TV2, TV3, TV, etc.), when needed. Each growth medium was supplemented by sodium pyruvate (Na-pyruvate; Fisher Scientific, Waltham, MA, USA), as indicated for each experiment. Anaerobic conditions were achieved by placing sterile mineral oil on top of the cultures, unless specified otherwise. Growth patterns over time were monitored by measuring the optical density at 600 nm (OD_600_) at 37 °C at 30 min intervals using a Bioscreen C growth curve analysis system (Growth Curves, Piscataway, NJ, USA). At least three independent experiments, each in quadruplicate, were performed. A representative result is presented in each relevant figure.

### 2.2. Mutant and Reporter Gene Fusion Construction

All mutants, including Δ*pfl* (SMU.402), Δ*pfl2* (SMU.493) and Δ*pflA* (SMU.1692), were created by using a PCR ligation mutagenesis approach [35], whereby each gene was deleted by replacing nearly all of the ORF with a non-polar kanamycin resistance marker (NPKm). Transformants were selected on BHI agar containing kanamycin, and double-crossover recombination into each gene was confirmed by PCR. To ensure that no mutations were introduced into flanking genes, the PCR fragments were sequenced. For conducting the GFP promoter reporter assays to monitor P*lrgA* activity, the P*lrgA-gfp* construct in the shuttle vector pDL278 [36] which was previously created [30], was transformed into the Δ*pfl*, Δ*pfl2* and Δ*pflA,* as well as Δ*lrgAB* mutants. As a control strain, the empty pDL278 plasmid was also transformed in these mutants.

### 2.3. Microplate Reporter Assay

To monitor *lrgAB* expression overgrowth, we used *S. mutans* strains harboring a P*lrgA-gfp* reporter fusion which was previously constructed [30]. Seed cultures of the reporter strains were diluted 1:50 into 175 µL fresh media in individual wells of a 96-well plate (black walls, clear bottoms; Corning) that was loaded into a Synergy microplate reader (BioTek, Winooski, VT, USA.) controlled by Gen5 software [26,27,30]. The optical density for growth curves and green fluorescence intensity for *lrgAB* expression measurement were monitored at 600 nm and 485 nm/520 nm (excitation/emission), respectively, at 30 min intervals for 18–24 h. The fluorescence of wild type harboring plasmid without the reporter gene fusion was subtracted from fluorescence readings of *S. mutans* strains harboring the P*lrgA-gfp* gene fusion. At least three independent replicates, each in triplicate, were performed. A representative result is presented in each relevant figure.

### 2.4. Measurement of Extracellular Pyruvate Levels

*S. mutans* UA159 wild type, Δ*pta*, Δ*ackA* and Δ*pta*Δ*ackA* mutants were grown in BHI medium at 37 °C in a 5% CO_2_ atmosphere. For time course measurements of extracellular pyruvate, excreted during growth, samples (300 µL) were taken at 1–2 h intervals and 150 µL was used to measure the OD_600_ in a spectrophotometer for monitoring growth. The rest of the sample (150 µL) was centrifuged for 2 min at 18,000× *g* to remove the cells, and pyruvate concentration of the supernatant was quantified with an EnzyChrom™ pyruvate assay kit (BioAssay Systems, Hayward, CA, USA) according to the manufacturer’s instructions. The level of extracellular pyruvate levels at certain time points (i.e., during early exponential growth and stationary phase) are predictable without measurement [26]. Thus, the pyruvate quantification was largely carried out with a particular focus on the samples taken at late-exponential and early-stationary phases in which *lrgAB* induction and pyruvate uptake through LrgAB occur. The results are average or representative of two independent replicates, each performed in duplicate.

## 3. Results

### 3.1. Low Glucose Dependence for Stationary Phase Pyruvate Uptake

**Figure 1:**
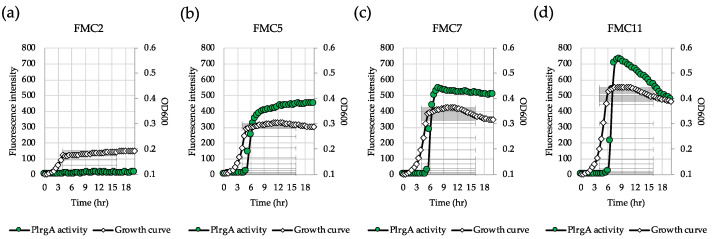
Low glucose dependence of P*lrgA* activity during growth in FMC medium. The P*lrgA-gfp* reporter strain in the *S. mutans* wild type background was grown in FMC medium, supplemented by 2 (**a**), 5 (**b**), 7 (**c**), and 11 mM (**d**) glucose. Relative *gfp* expression (green circle) and OD_600_ (white diamond) were monitored during growth on a plate reader (see Materials and Methods for details). The results are representative of three independent experiments.

*lrgAB* expression is highly responsive to glucose levels in *S. mutans* [29]. In order to identify the metabolic components related to *lrgAB* induction, we further explored how *lrgAB* responds to glucose levels lower than 11 mM, the concentration previously shown to allow strong stationary phase induction of *lrgAB* [26,30]. For this, we monitored *lrgAB* promoter activity by culturing the P*lrgA-gfp* strain in the wild type background [26,27,30] in FMC2, FMC5, FMC7 and FMC11. A chemically-defined FMC medium was recently reported to contain abundant acetate and potassium ions which are essential for *lrgAB* induction, consequently generating the highest fluorescent intensity (P*lrgA* activity) among the various media that were tested [27]. When the *gfp* reporter strain was cultivated in FMC2, no obvious *lrgAB* induction was observed at stationary phase, reaching OD_600_≈0.15 (Figure 1a). However, in FMC5, a sharp induction typical of *lrgAB* expression was observed at early stationary phase (Figure 1b), and further increases of glucose (7 mM and 11 mM) modestly elevated the overall induction level of *lrgAB* in early stationary phase (Figure 1c, d). Additionally, in FMC3, a basal induction level (approximately 30 fluorescence units) of *lrgAB* was observed (Supplementary Figure S1a). Thus, approximately 5 mM glucose seems to be required for a marked *lrgAB* induction when *S. mutans* is cultivated in FMC medium.

### 3.2. The Response of lrgAB to Extracellular Pyruvate is more Sensitive in Glucose-Limited Conditions

**Figure 2:**
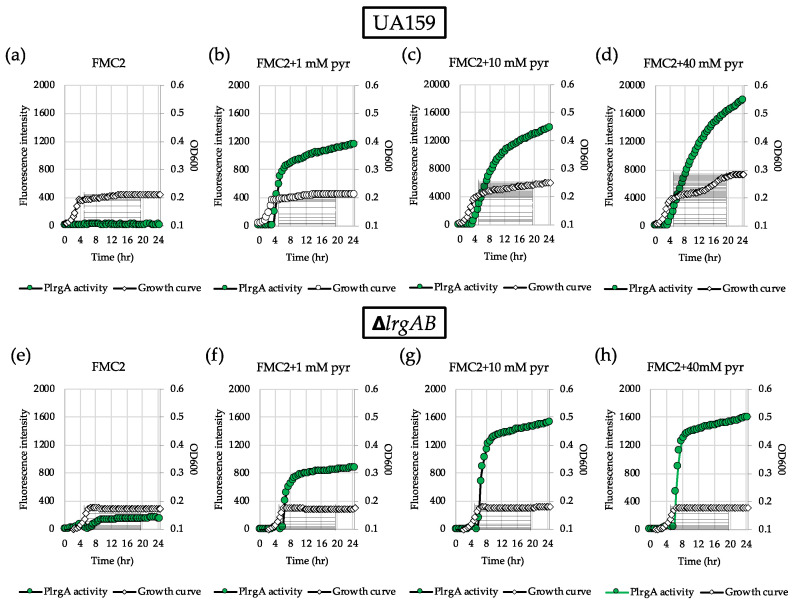
Change of P*lrgA* activity during growth of *S. mutans* UA159 wild type and Δ*lrgAB* mutant strains in FMC2 medium, supplemented by different concentrations of extracellular pyruvate. For measurement of P*lrgA* activation, the P*lrgA-gfp* reporter strain in the wild type (top panel) and Δ*lrgAB* (bottom panel) backgrounds was grown in FMC2 medium, supplemented by 0 (**a** and **e**), 1 (**b** and **f**), 10 (**c** and **g**) and 40 mM (**d** and **h**) of pyruvate (pyr). Relative *gfp* expression (green circle) and cell growth (white diamond) were monitored during growth on a plate reader (see Materials and Methods for details). The results are representative of three independent experiments.

**Figure 3:**
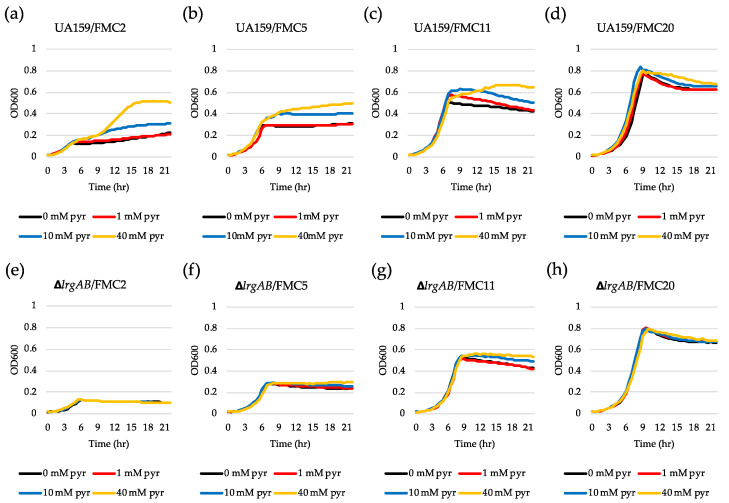
The effect of exogenously added pyruvate on the stationary phase of growth of *S. mutans* UA159 wild type and Δ*lrgAB* mutant strains. Wild type (**a**–**d**) and Δ*lrgAB* (**e**–**h**) strains were grown in FMC2 (a and e), FMC5 (b and f), FMC11 (c and g) and FMC20 (d and h), supplemented by different concentrations of pyruvate (0, 1, 10 and 40 mM). Optical density at 600 nm was monitored every 30 min at 37 °C using the Bioscreen C lab system. The results are representative of three independent experiments.

To further investigate why *lrgAB* was not induced when the cell was cultivated in FMC2, we wondered whether the cells are still responsive to extracellular pyruvate. For this, we added different concentrations of exogenous pyruvate to FMC2 at time of inoculation and monitored *lrgAB* expression overgrowth using the same *gfp* reporter (P*lrgA-gfp*) strain. No pyruvate control (Figure 2a) still exhibited no induction, as observed in Figure 1a. However, addition of 1 mM pyruvate to FMC2 remarkably elicited *lrgAB* expression, reaching approximately 1200 fluorescence units at 24 h (Figure 2b). Increasing the exogenous pyruvate concentration to 10 mM further elevated the *lrgAB* induction level by >10-fold (Figure 2c), compared to that observed with 1 mM pyruvate (Figure 2b). Interestingly, unlike what was observed in FMC11 supplemented by 40 mM pyruvate [26], the negative effect of high exogenous pyruvate concentrations on *lrgAB* expression was markedly alleviated in FMC2 supplemented by 40 mM pyruvate, without a reduction of *lrgAB* expression (Figure 2d). These results suggest that cells grown in FMC2 can more efficiently respond to exogeneous pyruvate, and the negative regulation of *lrgAB* by high concentrations of exogenous pyruvate can be alleviated by glucose limitation. We next evaluated how these observed responses of *lrgAB* expression to supplemented pyruvate impact the growth of the organism. For this, we monitored the growth of the *S. mutans* wild type strain in FMC2, supplemented with increasing concentrations of pyruvate (1, 10 and 40 mM) using a Bioscreen C plate reader. As shown in Figure 3a, supplemented pyruvate in FMC2, especially ≥10 mM, efficiently promoted stationary phase growth yields. A notable diauxic growth pattern was observed in wild type FMC2 cultures supplemented with 40 mM pyruvate, similar to that observed in Figure 2d, suggesting the utilization of pyruvate as a secondary carbon source. When this same experiment was repeated in FMC5, FMC11 and FMC20, the impact of exogenous pyruvate on stationary phase growth yields was lost as the glucose level increased in the medium (Figure 3b–d). In FMC20, previously reported to suppress *lrgAB* induction [29], added pyruvate had no obvious effect on stationary phase growth, although it appeared to alleviate stationary phase cell lysis (Figure 3c, d). These data suggest that uptake of extracellular pyruvate can be facilitated when cells grow in a glucose-limited environment. Given the correlation between overflowed pyruvate levels and *lrgAB* induction, the lack of induction in the presence of 2 mM glucose could be due to reduced pyruvate overflow.

### 3.3. LrgAB is Essential for Stationary Phase Pyruvate Uptake under Glucose-Limiting Growth Conditions

We previously reported that LrgAB is responsible for uptake and further utilization of excreted pyruvate at stationary phase [26]. Nevertheless, supplementation of exogenous pyruvate to FMC11 was still able to prolong the exponential growth of the Δ*lrgAB* mutant, as was also observed in the wild type strain, suggesting that inactivation of LrgAB does not completely block uptake of pyruvate [26]. However, no further explanation was provided in that study. In fact, when tested in FMC11 using the same *gfp* reporter strain, the induction level of *lrgAB* was about 2-fold higher in the Δ*lrgAB* background (Supplementary Figure S2b) than what was observed in the wild type background (Supplementary Figure S2a). Similar *lrgAB* induction trends were also observed during growth in FMC2 (Figure 2e) and FMC3 (Figure S1c), noticeably eliciting *lrgAB* expression at stationary phase. This suggests that lack of LrgAB could negatively influence stationary phase *lrgAB* induction, metabolically increasing pyruvate need. However, Figure 2f–h show that the response of *lrgAB* to extracellular pyruvate is remarkably reduced in Δ*lrgAB* mutant cells compared to those observed in the wild type background (Figure 2b–d) in FMC2, especially when grown in the presence of 10 and 40 mM pyruvate (Figure 2g,h, respectively), compared to those observed in the wild type background (Figure 2 c,d). When *ΔlrgAB* mutant cells were cultivated in FMC3, lack of LrgAB more evidently reduced the response of *lrgAB* to 1 mM exogenous pyruvate (Supplementary Figure S1d), compared to that observed in the wild type background (Supplementary Figure S1b). This suggests that *lrgAB* expression may not efficiently respond to extracellular pyruvate in the absence of LrgAB. Consistent with this observation, the Δ*lrgAB* mutant was unable to utilize exogenously added pyruvate in FMC2, as this strain did not display increased stationary phase growth yield or prolongation of exponential growth (Figure 3e). In FMC5, the effect of exogenously added pyruvate on the growth of the Δ*lrgAB* strain was slightly enhanced but no evidence was observed on growth/cell yield at stationary phase, although cell lysis was alleviated by added pyruvate (Figure 3f). The effect of added pyruvate in this Δ*lrgAB* strain was more evident in FMC11, moderately prolonging the exponential growth and alleviating cell lysis (Figure 3g). Again, no obvious effect was observed in FMC20, further supporting that high glucose levels abolish the stationary phase *lrgAB* induction and pyruvate uptake [26,29]. Collectively, these results highlight that LrgAB is essential for the stationary phase pyruvate uptake system when *S. mutans* cells are grown in a nutrient (glucose)-limiting condition, likely facilitating recovery from the starvation response itself and/or affecting cell death/lysis in response to starvation.

### 3.4. TV Medium Provides a More Favorable Environment for Response of lrgAB to Glucose Limitation

**Figure 4:**
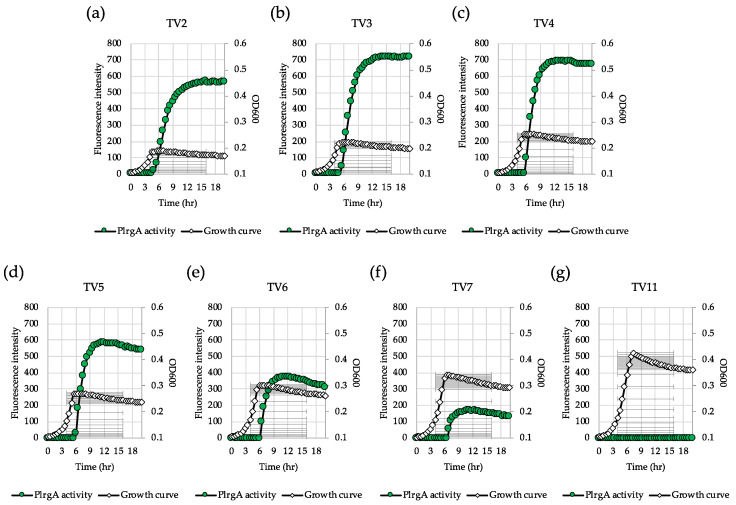
Low glucose dependence of P*lrgA* activity during growth in TV medium. The P*lrgA-gfp* reporter strain in the *S. mutans* wild type background was grown in TV medium, supplemented by 2 (**a**), 3 (**b**), 4 (**c**), 5 (**d**), 6 (**e**), 7 (**f**) and 11 mM (**g**) of glucose. Relative *gfp* expression (green circle) and OD_600_ (white diamond) were monitored during growth on a plate reader (see Materials and Methods for details). The results are representative of three independent experiments.

**Figure 5:**
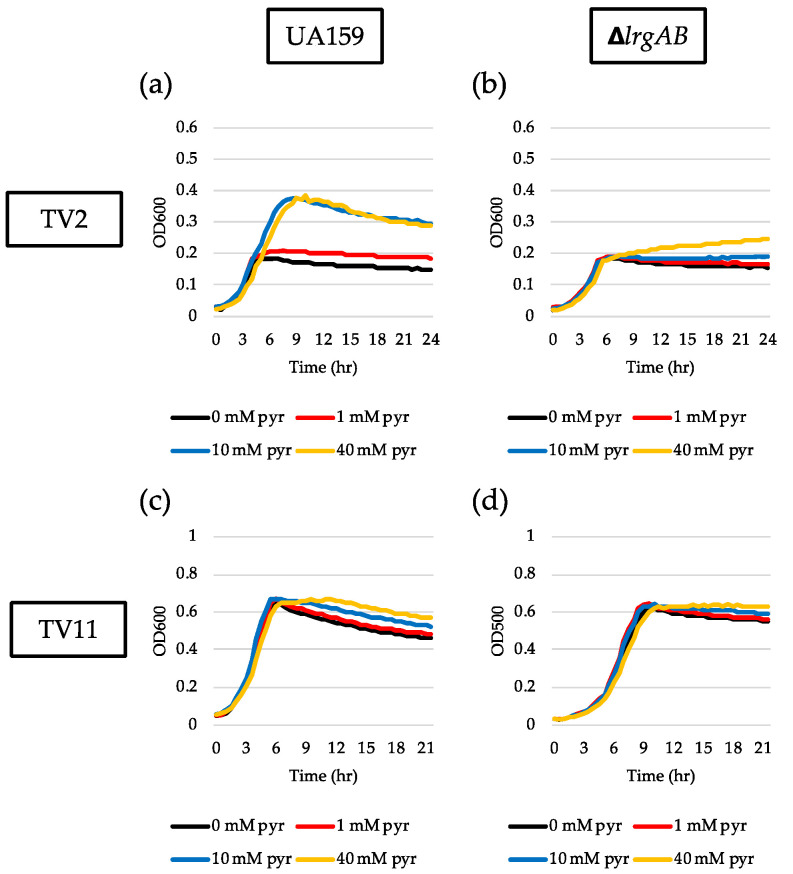
The effect of exogenously added pyruvate on the stationary phase of *S. mutans* UA159 wild type and Δ*lrgAB* mutant strains, grown in TV2 and TV11 media. Wild type (**a** and **c**) and Δ*lrgAB* (**b** and **d**) strains were grown in TV2 (**a** and **b**) and TV11 (**c** and **d**), supplemented by different concentrations of pyruvate (0, 1, 10 and 40 mM). Optical density at 600 nm was monitored every 30 min at 37 °C using the Bioscreen C lab system. The results are representative of three independent experiments.

Our recent study showed that the magnitude of *lrgAB* induction and pyruvate flux depends on the growth medium [27]. In this previous study, we demonstrated that stationary phase *lrgAB* induction and pyruvate uptake are completely blocked in TV11, suggesting that TV medium provides a different environment from FMC. We previously showed that TV medium primarily contains limited amounts of acetate and potassium ion which significantly affects the expression of genes, related to the Pta-AckA pathway, relative to FMC. Thus, we wondered whether LrgAB still plays a critical role in taking up pyruvate in glucose-limited TV medium. To test the idea, we technically recapitulated the above experiments in TV medium. In the GFP quantification assays for measurement of P*lrgA* activity, we first observed that *lrgAB* was remarkably induced at stationary phase when the reporter strain was cultivated in TV2 (Figure 4a), in contrast with that observed in FMC2 (Figure 1a). The induction level of *lrgAB* was about 25% further elevated in TV3 with growth enhancement of about 35% (Figure 4b), relative to that in TV2 (Figure 4a). However, further increases of glucose concentration negatively influenced *lrgAB* induction (Figure 4c–f), which was abolished in TV11, as reported recently [27]. This suggests that TV medium provides a more favorable condition for *lrgAB* induction than FMC under glucose limitation. A similar trend was also observed in the Δ*lrgAB* background (Supplementary Figure S3a–d), although the induction levels of *lrgAB* were about 50% lower than those observed in the wild type background (Figure 4). This suggests that lack of LrgAB positively influences stationary phase *lrgAB* induction in glucose-limited TV medium, which is in contrast with those observed in FMC (Figure 2a, e, Supplementary Figure S2). The effect of exogenously added pyruvate on the prolongation of exponential growth was more profound in TV2 (Figure 5a) than FMC2 (Figure 3a). But the effect was dramatically reduced in the Δ*lrgAB* strain (Figure 5b), similar to that observed in FMC2 (Figure 3e), suggesting that LrgAB is essential for stationary phase pyruvate uptake in glucose-limiting conditions regardless of growth medium (i.e., TV or FMC). As expected, exogenously added pyruvate had no marked effect on the stationary phase of wild type growth in TV11 (Figure 5c), which is consistent with our recently published results [27]. No obvious difference on growth was observed with exogenous pyruvate in the Δ*lrgAB* strain (Figure 5d). Therefore, these results suggest that *lrgAB* may more efficiently respond to external pyruvate in TV than FMC when the cell experiences glucose limitation, which could be due to a different pyruvate catabolic rate between two media.

### 3.5. Inactivation of the Pta-AckA Pathway Completely Inhibits Stationary Phase lrgAB Induction and Pyruvate Uptake

**Figure 6:**
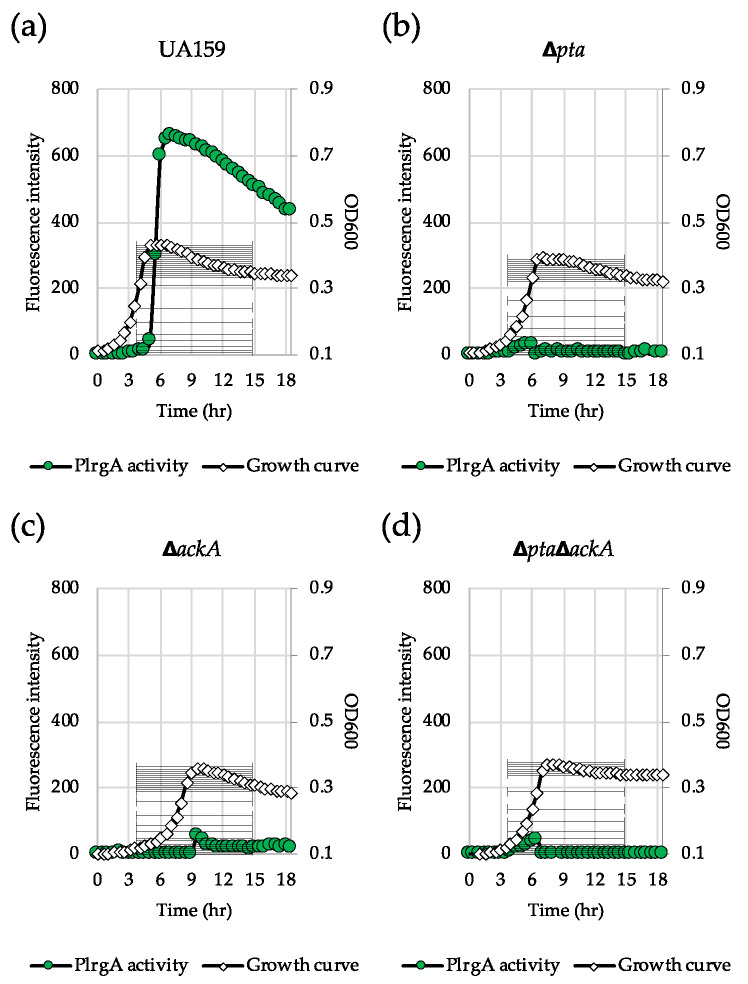
The contribution of the Pta-AckA pathway to stationary phase P*lrgA* activity. For measurement of P*lrgA* activation, the P*lrgA-gfp* reporter strain in the wild type UA159 (**a**), Δ*pta* (**b**), Δ*ackA* (**c**) and Δ*pta*Δ*ackA* (**d**) backgrounds, was grown in FMC11 medium. Relative *gfp* expression (green circle) and cell growth (white diamond) was monitored during growth on a plate reader (see Materials and Methods for details). The results are representative of three independent experiments.

**Figure 7:**
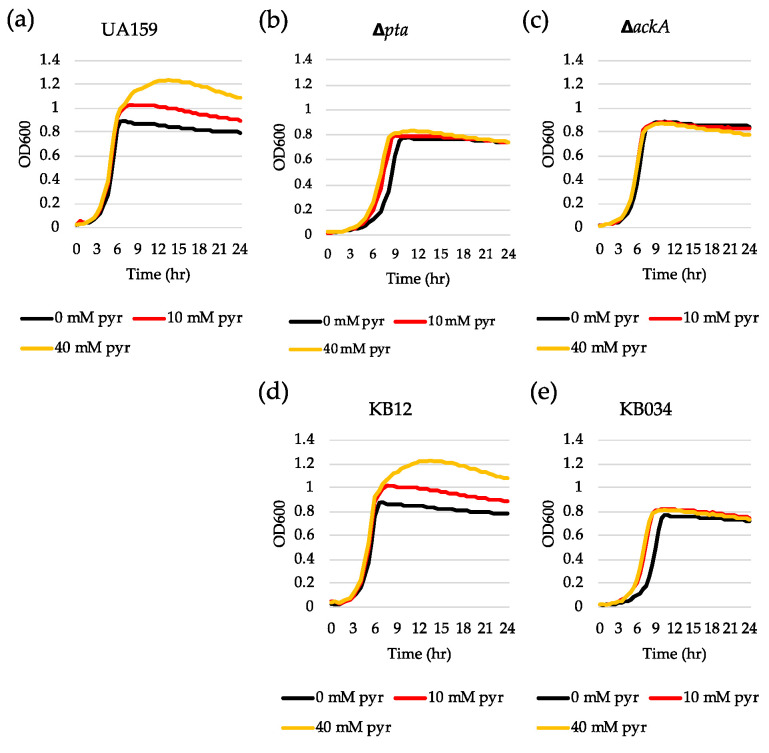
The effect of exogenously added pyruvate on the stationary phase of growth of *S. mutans* UA159 wild type and Pta-AckA pathway derivative strains. Wild type (**a**), Δ*pta* (**b**), Δ*ackA* (**c**), KB12 (*pta*-complemented, (**d**) and KB034 (*ackA*-complemented, (**e**) strains were grown in BHI, supplemented by different concentrations of pyruvate (0, 10 and 40 mM). Optical density at 600 nm was monitored every 30 min at 37 °C using the Bioscreen C lab system. The results are representative of three independent experiments.

In glucose-limited conditions, pyruvate is converted to acetyl coenzyme A (acetyl-CoA) by pyruvate formate lyase (Pfl) or pyruvate dehydrogenase complex (Pdh), depending on oxygen availability. Acetyl-CoA is then metabolized to acetate by the Pta-AckA pathway with the production of one molecule of ATP [37]. We recently revealed that the genes encoding the key enzymes (Pta, phosphate acetyltransferase and AckA, acetate kinase) of the Pta-AckA pathway were significantly upregulated during the transition to stationary phase in BHI and FMC11 (*lrgAB*-inducible) but not in TV11 (*lrgAB*-noninducible) [27]. Therefore, we assumed that the Pta-AckA pathway may participate in the regulation of *lrgAB*, especially in this glucose-limiting condition. To test this possibility, we transformed the same P*lrgA-gfp* reporter construct into the Δ*pta,* Δ*ackA* and Δ*pta*Δ*ackA* strains [38,39] and monitored the expression of *lrgAB* in FMC11, allowing the strongest stationary phase induction of *lrgAB* among the media tested [27,30]. Interestingly, inactivation of either *pta* (Figure 6b) or *ackA* (Figure 6c), or both genes (Figure 6d) almost completely inhibited expression of *lrgAB* at stationary phase, compared to that observed in the wild type background (Figure 6a). All mutant strains also exhibited a lower growth rate than the wild type strain in FMC11, suggesting that the Pta-AckA pathway is important for cell growth. More interestingly, the induction level of *lrgAB* was not elevated even by exogenously adding pyruvate to these mutant cultures (Supplementary Figure S4b–d), unlike what was observed in the wild type background (Supplementary Figure S4a), suggesting that *S. mutans* cells do not respond to external pyruvate in the absence of Pta or AckA. In accordance with these observations, exogenously added pyruvate had no obvious regrowth effect on the exponential or stationary phase of both Δ*pta* (Figure 7b) and Δ*ackA* (Figure 7c) strains, unlike what was observed in the wild type strain (Figure 7a). However, it did slightly enhance a growth rate of the Δ*pta* strain (Figure 7b). Similar results were obtained when tested in different media, including FMC11 (Supplementary Figure S5a-c) and TV3 (Supplementary Figure S5d-f), recently reported to be acetate-enriched and -limited, respectively. Thus, the lack of acetate by disruption of the Pta-AckA pathway does not seem to affect inhibited *lrgAB* induction and pyruvate uptake. When the *pta* mutation was complemented by producing Pta in a shuttle plasmid pDL278 [36,39], exogenously added pyruvate was normally utilized to prolong the exponential growth of the complemented strain (Figure 7d), as observed in the wild type strain (Figure 7a), further supporting the intimate linkage of the Pta pathway to stationary phase *lrgAB* induction and pyruvate uptake. However, no complementation was observed in the Δ*ackA* mutant strain [38], although the growth rate was slightly enhanced in the complemented strain (Figure 7e), suggesting that AckA activity may be subjected to additional metabolic regulation.

### 3.6. Inactivation of the Pta-AckA Pathway Modulates Pyruvate Flux

**Figure 8:**
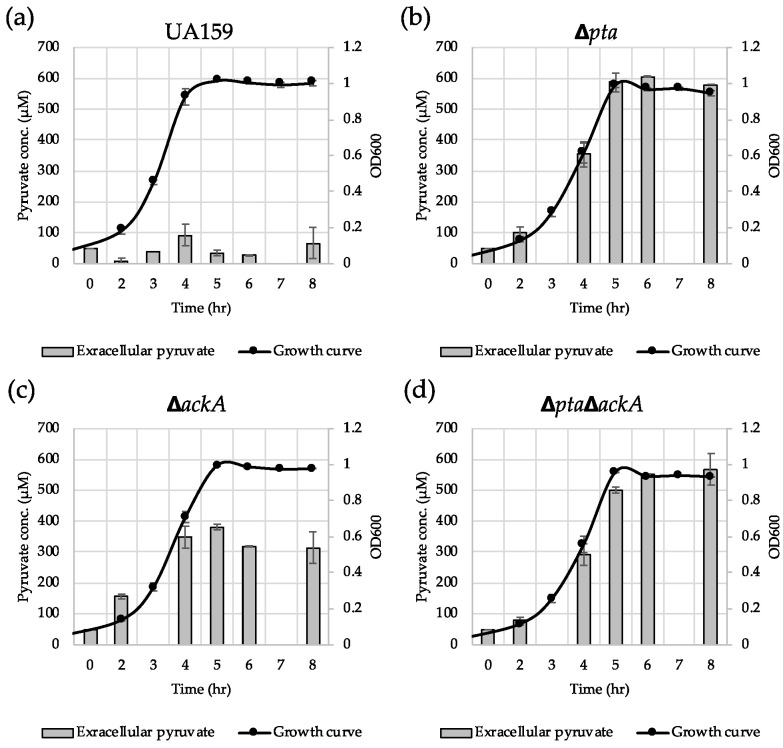
Measurement of extracellular pyruvate during growth of *S. mutans* UA159 wild type and Pta-AckA pathway derivative strains. Wild type (**a**), Δ*pta* (**b**), Δ*ackA* (**c**) and Δ*pta*Δ*ackA* (**d**) strains were grown in BHI. For time course measurements of extracellular pyruvate and growth, samples were taken at 1 or 2 h intervals (see Materials and Methods for details). The concentration of pyruvate was determined using an EnzyChrom™ pyruvate assay kit, and growth was measured by the optical density at 600 nm (OD_600_). Bars indicate the average concentration of extracellular pyruvate and a solid line with black circles indicates the corresponding growth curve. The results are an average of two independent experiments. Error bars = standard deviation.

To further elucidate how the Pta-AckA pathway effects *lrgAB* induction, we monitored the extracellular concentration of pyruvate during cultivation of the *S. mutans* wild type and Pta-AckA pathway-deficient strains in BHI. The wild type culture excreted approximately 93 µM of pyruvate at peak (late exponential phase) (Figure 8a), which is consistent with what was previously observed [26]. Somewhat surprisingly, the other mutant cultures excreted much higher levels of pyruvate during growth: approximately 600 µM for Δ*pta* (Figure 8b), approximately 380 µM for Δ*ackA* (Figure 8c), and approximately 550 µM for Δ*pta*Δ*ackA* (Figure 8d). Thus, considering the previously observed extracellular pyruvate concentration dependence of *lrgAB* induction [26], blockage of *lrgAB* induction by disruption of the Pta-AckA pathway does not seem to be due to excreted pyruvate. Also, in accordance with the observations in Figure 6 and Figure 7, reuptake of excreted pyruvate was almost completely blocked at the stationary phase of these mutant cultures (Figure 8b–d). Therefore, the data reinforce the direct linkage of the Pta-AckA pathway to pyruvate uptake LrgAB system and suggest that stationary phase pyruvate uptake may be determined by intracellular pyruvate consumption.

### 3.7. Pfl is Involved in the Regulation of lrgAB Expression

**Figure 9:**
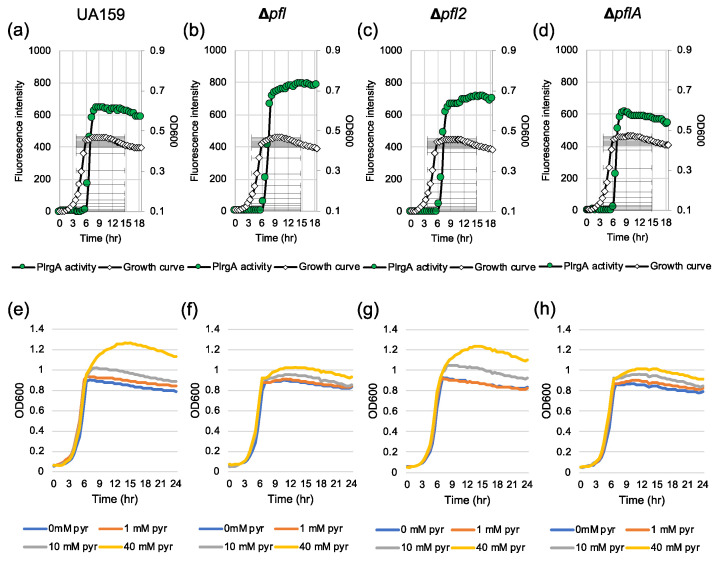
The contribution of the Pfl pathway to stationary phase P*lrgA* activity (**a**–**d**) and pyruvate uptake for regrowth (**e**–**h**). For measurement of P*lrgA* activation, the P*lrgA-gfp* reporter strain in the wild type UA159 (**a**), Δ*pfl* (**b**), Δ*pfl2* (**c**) and Δ*pflA* (**d**) backgrounds, was grown in FMC11 medium. Relative *gfp* expression (green circle) and cell growth (OD_600_; white diamond) were monitored during growth on a plate reader (see Materials and Methods for details). For measurement of growth in response to external pyruvate, wild type UA159 (**e**), Δ*pfl* (**f**), Δ*pfl2* (**g**) and Δ*pflA* strains were grown in BHI, supplemented by different concentrations of pyruvate (0, 10 and 40 mM). Optical density at 600 nm was monitored every 30 min at 37 °C using the Bioscreen C lab system. The results are representative of three independent experiments.

Given that the Pta-AckA pathway, responsible for the conversion of acetyl-CoA to acetate, plays a critical role in regulating stationary phase *lrgAB* induction and pyruvate uptake, we further assumed that the conversion of pyruvate to acetyl-CoA may also participate in the regulation of *lrgAB*. When glucose concentration is low, pyruvate is converted to acetyl-CoA and formate by Pfl (pyruvate formate lyase) or acetyl-CoA and CO_2_ by the Pdh (pyruvate dehydrogenase) complex, depending on the absence or presence of oxygen, respectively. In fact, our previous studies suggested a strong correlation between Pdh/Pfl and *lrgAB* regulation, because *pdhC* and *pfl* were also significantly upregulated at the early stationary phase, compared to that of the early exponential phase [27,28]. To test for a role of Pdh or Pfl in *lrgAB* regulation, we attempted to mutate the gene(s) encoding Pdh or Pfl. However, we failed to inactivate the *pdhC* gene or the entire *pdhDABC* operon, suggesting that these gene(s) may be essential for growth or genetic transformation. Three *pfl-*like genes, including *pfl* (SMU.402), *pfl2* (SMU.493) and *pflA* (SMU.1692), were identified in the *S. mutans* UA159 genome. Our previous microarray study showed that both *pfl* and *pfl2* genes were remarkably upregulated at the late exponential phase, compared to the early exponential phase [26,28]. The *pflA* gene is known to encode Pfl-activating enzyme, which is responsible for the conversion between active (O_2_-sensitive) and inactive (O_2_-resistant) forms of Pfl [40]. We successfully deleted all three genes and replaced them with a kanamycin resistant cassette, generating Δ*pfl*, Δ*pfl2* and Δ*pflA* strains. To monitor the expression of *lrgAB* throughout growth, we transformed the P*lrgA-gfp* reporter construct into each of these mutant strains. When the reporter strains were anaerobically grown in FMC11, the stationary phase induction level of *lrgAB* did not show a dramatic change in these mutant backgrounds compared to that in the wild type strain, although it was slightly elevated in the Δ*pfl* background (Figure 9a–d). However, the Δ*pfl* and Δ*pflA* mutant strains less efficiently utilized exogenously-added pyruvate at stationary phase (Figure 9f, h, respectively), compared to the wild type and Δ*pfl2* strains (Figure 9e,g, respectively). Although it is unclear from these data alone whether Pfl2 actually functions as a pyruvate formate lyase, these findings do suggest that *lrgAB* expression and pyruvate uptake are partially coordinated in the conversion of pyruvate to acetyl-CoA.

## 4. Discussion

In this present study, we demonstrate that the role of LrgAB as a pyruvate uptake system comes into its own when *S. mutans* encounters glucose limitation (at low mM levels), or experiences nutritional stress. This affords the organism a means to get out of stationary phase and contributes to the development of mature pathogenic biofilms and caries in the oral cavity when, from the bacterial point of view, it experiences prolonged periods of carbohydrate limitation. However, the magnitude and pattern of LrgAB activation depends on several external conditions, as observed in FMC- and TV-based media. Growth in TV medium appears to decrease the threshold of glucose induction of *lrgAB* because TV allows *lrgAB* induction or repression at lower glucose concentrations compared to FMC. Nevertheless, the finding that exogenously added pyruvate appeared to fuel a remarkable regrowth during stationary phase prolongation in both FMC and TV media containing 2 mM glucose, highlights that external pyruvate can be utilized as an alternative carbohydrate source for growth, especially under low glucose (nutrient) conditions. Similarly, we also found that stationary phase cell lysis could be alleviated by exogenously added pyruvate, suggesting that pyruvate can reactivate or stimulate the metabolic activity of *S. mutans* cells experiencing carbon starvation. It further suggests a potential connection between pyruvate regulation and cell death/lysis, hypothesized to be induced by the Cid/Lrg system, including LrgAB and its partner operon CidAB [29]. In fact, such a role of pyruvate as a metabolic stimulator has been reported in many pathogens, including *Campylobacter jejuni, E. coli, Lactobacillus acetotolerans, Legionella pneumophila, Salmonella typhi, Shigella flexneri, Shigella sonnei*, *Vibrio cholerae* and *Yersinia pestis* [41,42,43,44,45,46,47,48,49]. Although the precise underlying mechanisms are still unclear, these bacteria have been reported to enter the viable but non-culturable (VBNC) state in response to adverse environmental conditions, such as high/low temperature, osmotic stress, oxidative stress and starvation [6,46]. They could be resuscitated to a growing state by addition of pyruvate [18,50,51]. Therefore, we hypothesize that pyruvate plays an intricate role not only as a key metabolic intermediate of glycolysis but also as a growth-promoting molecule that could simulate metabolic activity, consequently enhancing the capacity of the organism to survive and persist against unfavorable environments. Considering that pyruvate could also provide protection against oxidants, which in turn would be less stress on the cells, inhibition/alleviation of cell lysis could be unrelated to pyruvate metabolism. 

It may not be surprising that the removal of the major acetate production pathway encoded by Pta (phosphotransacetylase) and AckA (acetate kinase) led to the blockage of *lrgAB* expression and subsequent pyruvate uptake, because acetate was recently reported to contribute to the induction of *lrgAB* to stationary phase [27]. A more interesting finding is that both the Δ*pta* and Δ*ackA* mutant strains became insensitive to exogenous pyruvate. A simple explanation for this could be that disruption of the Pta-AckA pathway could decelerate pyruvate consumption, accumulating intracellular pyruvate levels and consequently increasing the excretion of pyruvate as an overflow metabolite. It is also possible that inhibition of *lrgAB* induction in the Pta-AckA-deficient strains could also be due to increased acetyl-CoA levels [38]. Accordingly, our previous microarray experiment demonstrated that the genes responsible for the conversion of pyruvate to acetyl-CoA (*pfl2* and *pdhA*) were significantly downregulated in these mutant strains compared to the wild type strain [38]. Either way, these findings suggest that the alteration of pyruvate catabolic activity could affect the capacity of *S. mutans* cells to take up extracellular pyruvate, consequently affecting the degree of cell lysis and death at stationary phase. Given that inactivation of the Pta-AckA pathway could lead to redirection of the carbon flow into multiple metabolic pathways with changes in the levels of key metabolites including ATP, NAD^+^, NADH and other organic acids [52,53], the trend of *lrgAB* induction and pyruvate flux may be a result of the highly complicated regulatory behavior at the central pyruvate node, even with a possible involvement of glycolytic metabolites between glucose and pyruvate. In this study, we also showed that disruption of the Pfl pathway resulted in a moderate inhibition of pyruvate uptake at stationary phase which is consistent with the observed upregulation in expression of *pfl* at the late exponential phase compared to the early exponential phase [28]. This suggests that the Pfl pathway is also linked, at least in part, to the stationary phase response through LrgAB activity, even in the absence of oxygen. However, given the dramatic elevation of *pdh* expression at the late exponential phase compared to the early exponential phase [26,28] and complete inhibition of stationary phase *lrgAB* expression and pyruvate uptake by disruption of the Pta-AckA pathway, the majority of the carbon flux is likely channeled through the Pdh and Pta-AckA branch, which may determine the need of the cell to take up external pyruvate through LrgAB. Our previous microarray data also show that even by lack of Pta and AckA, the expression level of *ldh* which encodes lactate dehydrogenase and is responsible for the conversion of pyruvate to lactate, was not elevated [38]. This suggests that excess pyruvate, accumulated by disruption of the Pta-AckA pathway, would be overflowed without forwarding to the Ldh pathway under glucose limitation. Therefore, stationary phase *lrgAB* expression and pyruvate uptake seem to be primarily coordinated by continuous and rapid redirection of intracellular flux at the pyruvate node in response to changing environments, aerobically and nutritionally.

Another linkage between the Pta-AckA pathway and pyruvate uptake regulation may be related to the extracellular level of acetate, the major end product of the Pta-AckA pathway which is excreted as another overflow metabolite during growth to eliminate extra redox potential when glucose is no longer oxidized to CO_2_. We recently demonstrated that external acetate can enhance stationary phase *lrgAB* induction and inhibit growth in excess [27], suggesting that acetate may mediate the response of *lrgAB* to the stationary phase, as well as cell lysis and death. Although the disruption of the Pta-AckA pathway inhibits acetate production, lack of acetate does not seem to directly result in the observed dramatic reduction of *lrgAB* induction and pyruvate uptake, because the inhibitory effect of an inactive Pta-AckA pathway on *lrgAB* induction was observed in both FMC11 (acetate-enriched) and TV3 (acetate-limited). Nevertheless, the fact that, like the LrgAB system [30,31], the Pta-AckA pathway is also tightly regulated by CcpA and CodY [54], implicates that the pyruvate uptake and acetate production systems may be linked in a metabolically complex manner which warrants further study.

## 5. Conclusions

In conclusion, this current study identifies a major metabolic route for stationary phase *lrgAB* and pyruvate regulation and demonstrates that pyruvate can be utilized for growth as the secondary carbon source and potentiate alleviation of cell lysis at the stationary phase. An increase of external pyruvate levels increases both pyruvate uptake rate and intracellular pyruvate level [26,55]. Pyruvate may have activated certain metabolic pathways for energy generation and growth stimulation. Thus, further studies of these aspects will expand our understanding of the mechanisms for how *S. mutans* cells adapt to adverse environments such as nutritional starvation and oxidative condition, and consequently develop pathogenic mature biofilms (caries), especially through pyruvate metabolism and uptake.

## Supplementary Materials

The following are available online at https://www.mdpi.com/2076-2607/8/6/846/s1, Table S1: Bacteria strains and plasmids used in this study, Figure S1: Change of P*lrgA* activity during growth of wild type and Δ*lrgAB* mutant strains in FMC3 medium with or without 1 mM exogenous pyruvate, Figure S2: Change of P*lrgA* activity during growth of wild type and Δ*lrgAB* mutant strains in FMC11 medium, Figure S3: Changes of P*lrgA* activity during growth in TV medium containing low concentrations of glucose in the Δ*lrgAB* background, Figure S4: The response of P*lrgA* activity to 1 mM pyruvate in FMC 11 medium by disruption of the Pta-AckA pathway, Figure S5: The effect of exogenously added pyruvate on the stationary phase of *S. mutans* UA159 wild type, Δ*pta*, and Δ*ackA* strains, grown in FMC11 and TV3.
