# Supplementary material for: The Pta-AckA Pathway Regulates LrgAB-Mediated Pyruvate Uptake in Streptococcus mutans
Source: Microorganisms. 2020 Jun 4;8(6):846. doi: 10.3390/microorganisms8060846 (PMC7355876; doi:10.3390/microorganisms8060846)
Supplement: Supplementary file 1 [file microorganisms-08-00846-s001.pdf]

Table S1. Bacterial strains and plasmids used in this study.

Stains and plasmids	Genotype or relevant characteristics	Source
Strains		
UA159	Wild type	Laboratory stock
Δ*lrgAB*	Δ*lrgAB*::ΩKm^r	(29)
Δ*pta*	Δ*pta*::NPKm^r	(39)
Δ*ackA*	Δ*ackA*::NPKm^r	(38)
Δ*pta*Δ*ackA*	Δ*pta*::NPEm^r/Δ*ackA*::NPKm^r	(38)
KB12	Δ*pta* carrying pDL278::*pta* (*pta*-complemented strain)	(39)
KB034	Δ*ackA* carrying pDL278::*ackA* (*ackA*-complemented strain)	(38)
UA159/P*lrgA-gfp*	UA159 carrying *gfp* gene fusion to *lrgA* promoter	(30)
UA159/pDL278	UA159 carrying pDL278	(30)
Δ*lrgAB*/P*lrgA-gfp*	Δ*lrgAB* carrying *gfp* gene fusion to *lrgA* promoter	This study
Δ*lrgAB*/pDL278	Δ*lrgAB* carrying pDL278	This study
Δ*pta*/P*lrgA-gfp*	Δ*pta* carrying *gfp* gene fusion to *lrgA* promoter	This study
Δ*pta*/pDL278	Δ*pta* carrying pDL278	This study
Δ*ackA*/P*lrgA-gfp*	Δ*ackA* carrying *gfp* gene fusion to *lrgA* promoter	This study
Δ*ackA*/pDL278	Δ*ackA* carrying pDL278	This study
Δ*pta*Δ*ackA*/P*lrgA-gfp*	Δ*pta*Δ*ackA* carrying *gfp* gene fusion to *lrgA* promoter	This study
Δ*pta*Δ*ackA*/pDL278	Δ*pta*Δ*ackA* carrying pDL278	This study
Plasmids		
pDL278	*E. coli-Streptococcus* shuttle vector, Sp^r	(36)
pDL278::P*lrgA-gfp*	pDL278 carrying *gfp* gene fusion to *lrgA* promoter	(30)

Em^r, erythromycin resistant cassette; Km^r, kanamycin resistant cassette; Sp^r, spectinomycin resistant cassette; NP, nonpolar; Ω, polar.

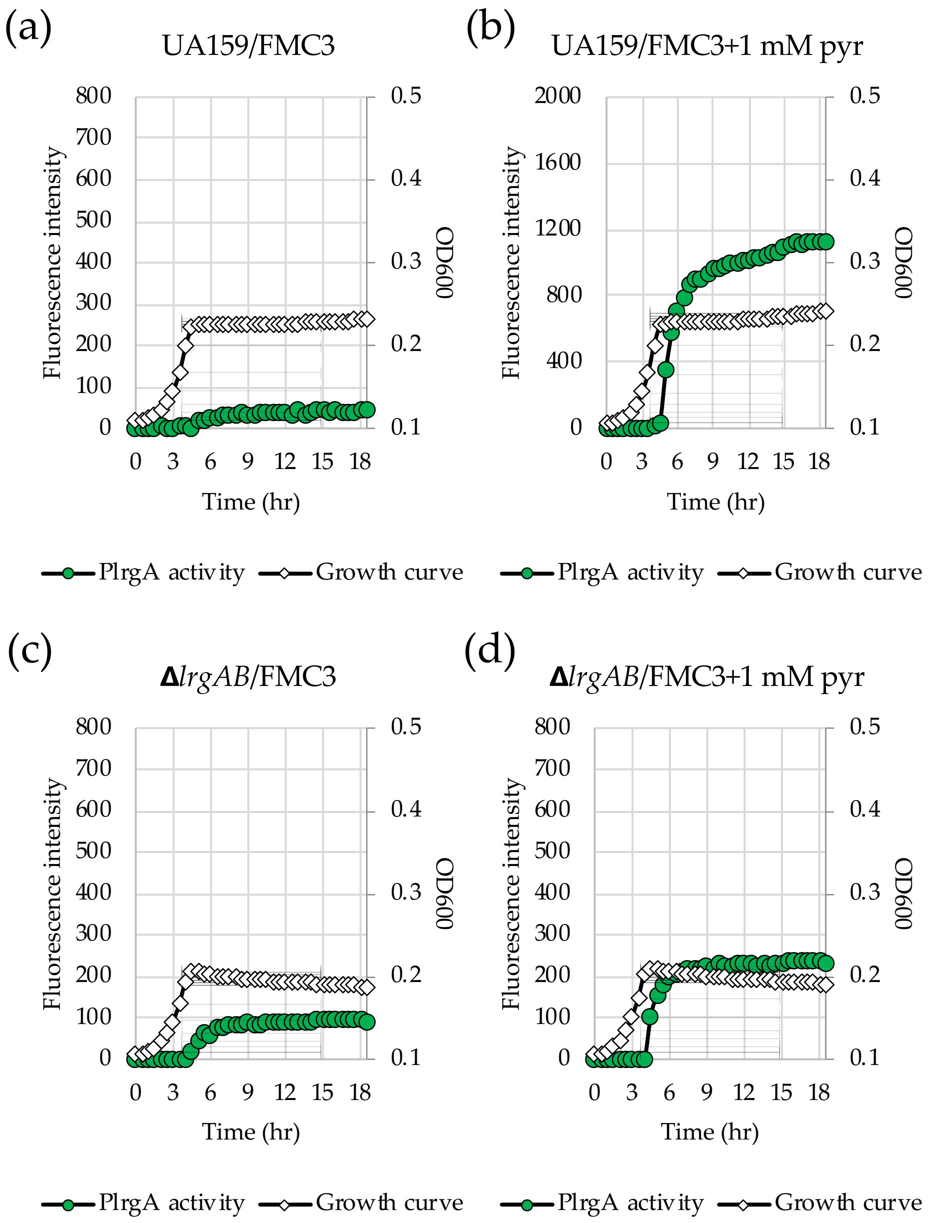

Figure S1. Change of P*lrgA* activity during growth of wild type and Δ*lrgAB* mutant strains in FMC3 medium with or without 1 mM exogenous pyruvate

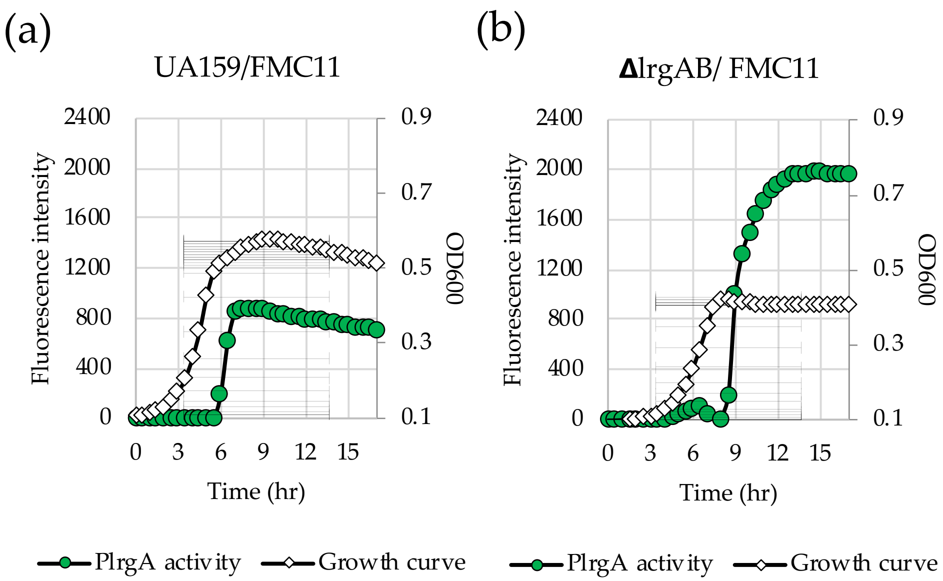

Figure S2. Change of P*lrgA* activity during growth of wild type and Δ*lrgAB* mutant strains in FMC11 medium.

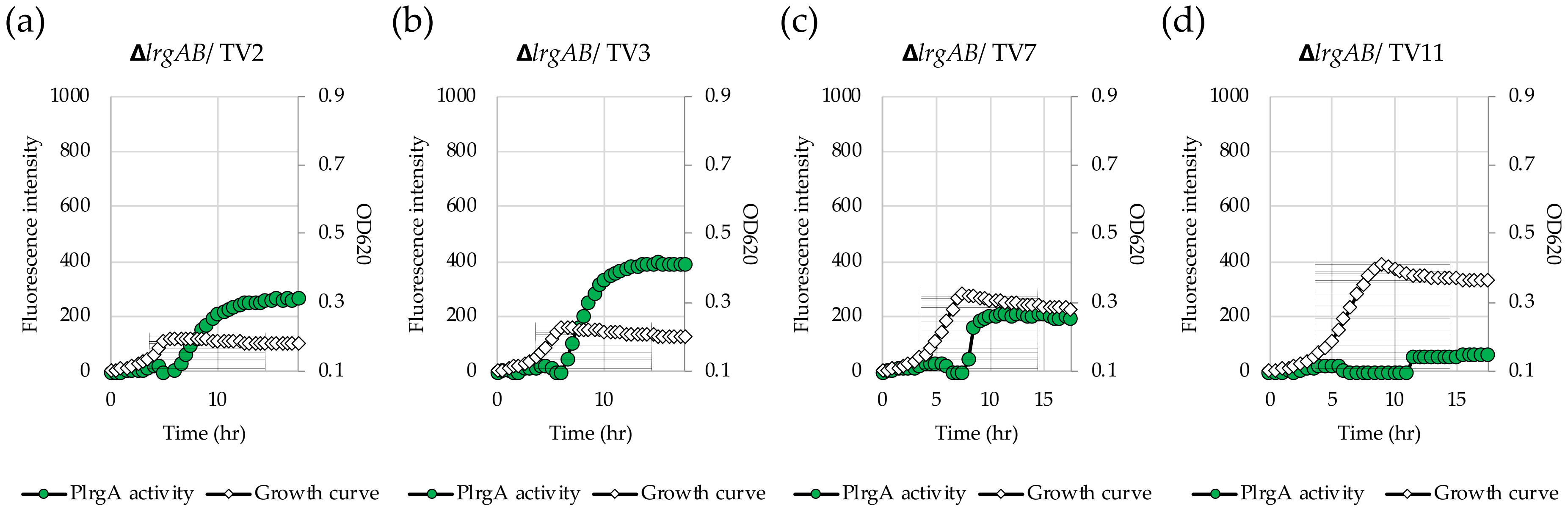

Figure S3. Changes of P*lrgA* activity during growth in TV medium containing low concentrations of glucose in the **Δ***lrgAB* background.

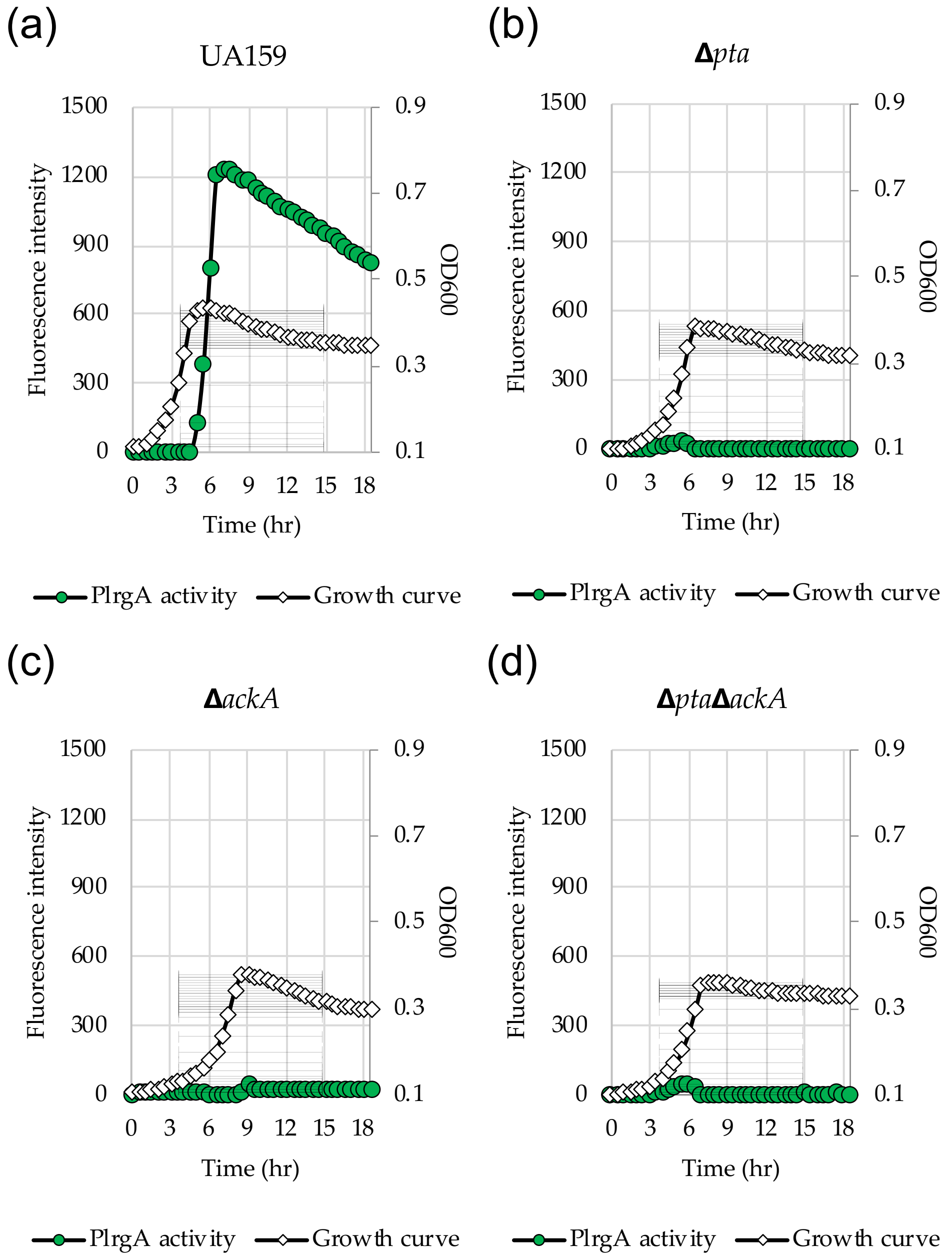

Figure S4. The response of P*lrgA* activity to 1 mM pyruvate in FMC 11 medium by disruption of the Pta-AckA pathway.

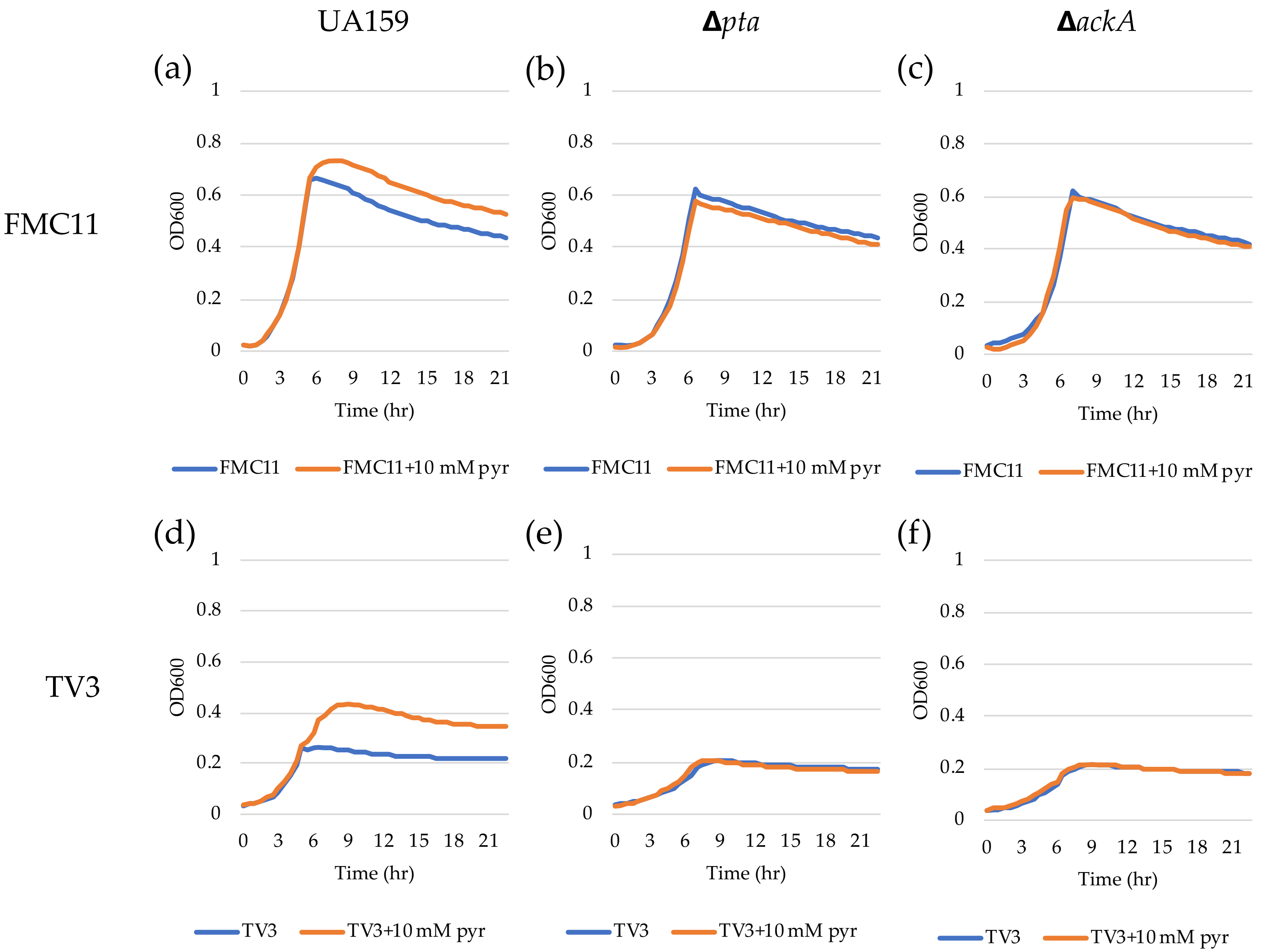

Figure S5. The effect of exogenously added pyruvate on the stationary phase of *S. mutans* UA159 wild type, Δ*pta* and Δ*ackA* strains, grown in FMC11 and TV3.
